# MTUS1 is a promising diagnostic and prognostic biomarker for colorectal cancer

**DOI:** 10.1186/s12957-022-02702-2

**Published:** 2022-08-13

**Authors:** Lin-Yao Cheng, Mao-sen Huang, Hua-Ge Zhong, Hai-Ming Ru, Si-Si Mo, Chun-Yin Wei, Zi-Jie Su, Xian-Wei Mo, Lin-Hai Yan, Wei-Zhong Tang

**Affiliations:** 1 Department of Gastrointestinal Surgery, Guangxi Medical University Cancer Hospital, Nanning, 530021 Guangxi Zhuang Autonomous Region China; 2 Guangxi Clinical Research Center for Colorectal Cancer, Nanning, 530021 Guangxi Zhuang Autonomous Region China; 3 Guangxi Key Laboratory of Colorectal Cancer Prevention and Treatment, Nanning, 530021 Guangxi Zhuang Autonomous Region China

**Keywords:** Colorectal cancer, Biomarker, Immune infiltration, MTUS1, Diagnosis, Prognosis

## Abstract

**Background:**

The morbidity and mortality of colorectal cancer (CRC) remain high, posing a serious threat to human life and health. The early diagnosis and prognostic evaluation of CRC are two major challenges in clinical practice. MTUS1 is considered a tumour suppressor and can play an important role in inhibiting cell proliferation, migration, and tumour growth. Moreover, the expression of MTUS1 is decreased in different human cancers, including CRC. However, the biological functions and molecular mechanisms of MTUS1 in CRC remain unclear.

**Methods:**

In the present study, data from The Cancer Genome Atlas (TCGA) database were analysed using R statistical software (version 3.6.3.) to evaluate the expression of MTUS1 in tumour tissues and adjacent normal tissues using public databases such as the TIMER and Oncomine databases. Then, 38 clinical samples were collected, and qPCR was performed to verify MTUS1 expression. We also investigated the relationship between MTUS1 expression and clinicopathological characteristics and elucidated the diagnostic and prognostic value of MTUS1 in CRC. In addition, the correlation between MTUS1 expression and immune infiltration levels was identified using the TIMER and GEPIA databases. Furthermore, we constructed and analysed a PPI network and coexpression modules of MTUS1 to explore its molecular functions and mechanisms.

**Results:**

CRC tissues exhibited lower levels of MTUS1 than normal tissues. The logistic regression analysis indicated that the expression of MTUS1 was associated with N stage, TNM stage, and neoplasm type. Moreover, CRC patients with low MTUS1 expression had poor overall survival (OS). Multivariate analysis revealed that the downregulation of MTUS1 was an independent prognostic factor and was correlated with poor OS in CRC patients. MTUS1 expression had good diagnostic value based on ROC analysis. Furthermore, we identified a group of potential MTUS1-interacting proteins and coexpressed genes. GO and KEGG enrichment analyses showed that MTUS1 was involved in multiple cancer-related signalling pathways. Moreover, the expression of MTUS1 was significantly related to the infiltration levels of multiple cells. Finally, MTUS1 expression was strongly correlated with various immune marker sets.

**Conclusions:**

Our results indicated that MTUS1 is a promising biomarker for predicting the diagnosis and prognosis of CRC patients. MTUS1 can also become a new molecular target for tumour immunotherapy.

## Introduction

Colorectal cancer (CRC) is a major cause of cancer-related morbidity and mortality worldwide [1]. Most CRC deaths are caused by primary tumours invading and metastasizing to other tissues [2]. Recently, the continuous development of treatment paradigms, especially clinical applications of targeted therapies and immunotherapies, has greatly improved the prognosis of CRC patients [3–5]. However, some patients cannot derive a benefit from these therapies. For example, various patients can develop resistance to targeted anticancer drugs during clinical applications, representing a major challenge for these therapeutic approaches [6], in addition to a low response rate and immune-related adverse effects [7]. Additionally, the exact immune-related mechanisms of CRC remain to be elucidated. CRC incidence rates are also increasing, and the age distribution of patients tends to be younger [8]. However, when CRC is detected in the early stages, patients usually have a good prognosis. Nevertheless, most patients are diagnosed in mid to late stages, mostly due to the lack of specific clinical symptoms and effective diagnostic methods at early stages. Therefore, reliable diagnostic markers and immune-related therapeutic targets for CRC patients have become a top priority.

The microtubule-associated scaffold protein 1 (MTUS1) gene is located on the 8p22 chromosomal region and consists of 17 exons. MTUS1 encodes several proteins with different functional properties, including ATIP1, ATIP3 (ATIP2, ATIP3a, and ATIP3b), and ATIP4 [9]. ATIP1 and ATIP3 mediate cellular apoptotic mechanisms and interfere with growth-promoting signals, thereby affecting the occurrence and progression of cancers [10–12]. Additionally, MTUS1 is involved in the pathological process of cardiac hypertrophy and SLE-like lymphoproliferative diseases [13]. Previous studies have also suggested that MTUSI is downregulated in multiple cancers, including CRC. MTUS1 is also associated with poor prognosis in patients with lung adenocarcinoma, gastric cancer, renal cell carcinoma, gallbladder carcinoma, salivary adenoid cystic carcinoma, and oral tongue squamous cell carcinoma [10, 14–17].

Nevertheless, the mRNA expression level and prognostic potential of MTUS1 in CRC remain unexplored due to its connection with tumour-infiltrating lymphocytes (TILs). Therefore, in the present study, we systematically analysed the expression of MTUS1 and its correlation with the clinicopathological characteristics and prognosis of CRC patients for the first time. We constructed a protein interaction network and performed enrichment analyses to reveal the molecular functions and underlying regulatory mechanisms of MTUS1. Moreover, we investigated the relationship between MTUS1 and tumour-infiltrating immune cells and markers. Our results demonstrated the prognostic and diagnostic value of MTUS1 and clarified its tumour suppressor effects in CRC. Overall, MTUS1 can be a novel diagnostic and prognostic marker and an immune cell infiltration predictor for CRC patients. Finally, these results might also provide new ideas for CRC clinical diagnoses and immunotherapies.

## Materials and methods

### Data acquisition

Datasets with gene expression profiles and paired clinical information of CRC patients were retrieved from the TCGA database and included 698 tissues (51 normal and 647 tumour tissues). Then, we excluded patients with missing data, such as age, overall survival time, TNM stage, and distant metastasis. Finally, 643 patients with complete clinical information were used in univariate and multivariate regressions and immune infiltration analyses. Additionally, the GSE23878 colorectal microarray dataset was retrieved from the GEO public bioinformatics database (www.ncbi.nlm.nih.gov/geo) to validate the expression of MTUS1.

### Collection of clinical samples

To further validate the mRNA expression level of MTUS1, we collected 38 primary CRC tissues and paired normal tissues at the Guangxi Medical University Cancer Hospital from September 2021 to December 2021.

### Total RNA extraction and quantitative real-time PCR analysis

Total RNA was isolated from the clinical samples using TRIzol reagent (TaKaRa, Japan) according to the manufacturer’s protocol. The RNA samples were reverse transcribed into cDNA using the PrimeScript™ RT Reagent Kit (TaKaRa) and then subjected to PCR amplification. Real-time quantitative PCR was performed with SYBR Green fluorescence detection in a quantitative PCR thermal cycler at 95 °C for 2 min, followed by a two-step amplification program (15 s denaturation at 95 °C, 1 min annealing/extension at 60 °C) repeated for 40 cycles (ABI 7500, Applied Biosystems). Each reaction was prepared using 2 μL of cDNA, 10 μL of Eastep™ qPCR Master Mix (Promega, LS2062), 0.4 μL each of the forward and reverse primers, and 7.2 μL of DNase-free water in a total volume of 20 μL. The relative expression of MTUS1 was determined according to DeltaDeltaCt with normalization to GAPDH expression. The primer sequences used for qRT–PCR were as follows: MTUS1, forward: 5′- TTGACAAATTGAAGCGTTTCCAG-3′, reverse: 5′- CTGCCTTGAGATTGCCATGTG-3′; and GAPDH, forward: 5′- CCAGAACATCATCCCTGCCTCTACT-3′, reverse: 5′-GGTTTTTCTAGACGGCAGGTCAGGT-3′.

### Oncomine database analysis

The Oncomine database (http://www.oncomine.org) contains 715 datasets and 86,733 tumour and normal samples. Here, Oncomine was used to analyse MTUS1 mRNA expression dissimilarities between cancer and normal tissues. The thresholds were set as follows: *p value* < 1E-4; fold change (FC): 2; gene rank: all; and data type: mRNA.

### TIMER analysis

TIMER (https://cistrome.shinyapps.io/timer/) is a comprehensive web tool that can be used to analyse immune infiltrates from multiple perspectives in human cancers. In the present study, TIMER was used to determine the correlation between the expression of MTUS1 and the abundance of tumour-infiltrating immune cells, including B cells, CD4+ T cells, CD8+ T cells, neutrophils, macrophages, and dendritic cells. We also compared tumour infiltration levels among tumours with different somatic copy number alterations in MTUS1. The different expression levels of MTUS1 between tumour and adjacent normal tissues were also analysed using TIMER.

### GEPIA

GEPIA (http://gepia.cancer-pku.cn/index.html) is an online analysis tool for RNA sequencing (RNA-seq) data based on TCGA and GTEx data. In the present study, GEPIA was used to analyse the correlation between MTUS1 and the gene markers of different tumour-infiltrating immune cells. Spearman’s rank correlation coefficient was used to determine the significance of correlations.

### STRING analysis

The STRING (https://string-db.org/) database was used to predict protein–protein interactions (PPIs) and build a network of functionally related proteins.

### Data processing and statistical analysis

Differences in MTUS1 mRNA expression levels in nonpaired samples were compared using Wilcoxon rank-sum tests. In paired samples, the Wilcoxon signed-rank test was used to estimate expression differences. The median value of MTUS1 mRNA expression was set as the cut-off value to stratify patients into groups with high and low expression of MTUS1. The Kruskal–Wallis and *χ*^2^ tests were used to assess the correlation between MTUS1 expression and clinicopathological features. The Kaplan–Meier method was used to assess OS and DSS distributions between the high and low MTUS1 groups. Differences between survival curves were compared by the log-rank test. Univariate and multivariate Cox proportional hazards regression models were used to evaluate the effects of MTUS1 mRNA expression and clinicopathologic characteristics on the OS of CRC patients. Then, we constructed a nomogram based on the Cox proportional hazards regression models to determine independent prognostic factors, and calibration curves were used to assess the predictive efficacy of the model. To investigate the potential diagnostic value of MTUS1, we determined the area under the receiver operating characteristic (ROC) curve (AUC). Next, to better understand the function of MTUS1, we identified 50 coexpressed genes with the highest positive and negative correlations. Subsequently, Gene Ontology (GO) and Kyoto Encyclopedia of Genes and Genomes (KEGG) pathway enrichment analyses were performed to annotate the functions of the coexpressed genes. Furthermore, to explore the role of MTUS1 in colorectal cancer, we performed single-sample gene set enrichment analysis (ssGSEA) to investigate the impact of MTUS1 on various tumour-infiltrating immune cells. Associations between MTUS1 and the infiltration levels of different immune cells were examined using Spearman’s correlation tests and *p value*s. R 3.6.3 software was used for statistical analyses, and a *p value* < 0.05 was considered statistically significant.

## Results

### MTUS1 mRNA expression levels in different types of human tumours

**Fig. 1:**
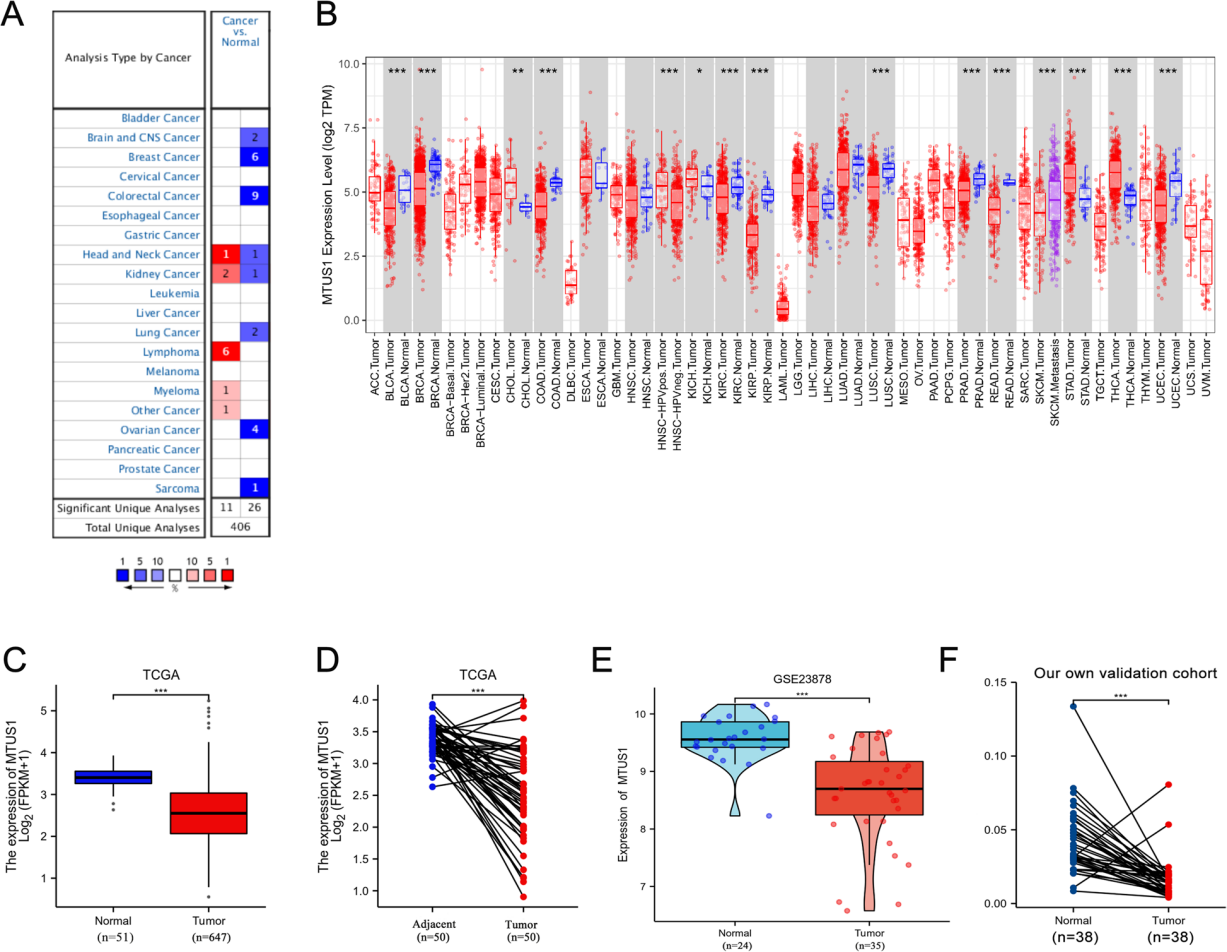
MTUS1 expression in different cancer types: **A** MTUS1 expression levels in different cancer tissues compared with normal tissues in the Oncomine database. Nine studies confirmed that MTUS1 was downregulated in colorectal cancer. **B** MTUS1 expression levels in different cancer types in the TIMER database (*p < 0.05, **p < 0.01, ***p < 0.001). This result was consistent with the Oncomine database (**A**). **C** Expression of MTUS1 in tumour and normal tissues from the TCGA database. **D** Comparison of MTUS1 expression in 50 pairs of tumour and adjacent tissues. **E**, **F** MTUS1 expression in the GSE13507 array and our recruited cohort

First, we used the Oncomine database to analyse MTUS1 expression levels in different human tumour tissues and healthy tissues. The results showed that the expression of MTUS1 was higher in head and neck cancer, kidney cancer, lymphoma, myeloma, and other cancer tissues than in normal tissues. Meanwhile, in brain and CNS cancers, breast cancer, CRC, head and neck cancer, kidney cancer, ovarian cancer, sarcoma, and lung cancer, MTUS1 mRNA expression was lower than that in adjacent normal controls (Fig. 1A). Furthermore, we used the TIMER database to examine which cancers have differences in MTUS1 mRNA expression levels. We found that the expression of MTUS1 in CHOL, KICH, STAD, and THCA was significantly higher than that in normal tissues. In contrast, MTUS1 was expressed at lower levels in nine cancer types (BLCA, BRCA, COAD, KIRC, KIRP, LUSC, PRAD, READ, and UCEC) than in normal controls (Fig. 1B). Then, we analysed the MTUS1 RNA expression data of CRC and normal tissues from the TCGA database (September 2021) using the Wilcoxon rank-sum test. CRC tissues exhibited significantly lower MTUS1 mRNA expression levels than normal tissues (Fig. 1C). Additionally, the levels of MTUS1 in 50 paired CRC and adjacent nontumorous tissues were compared using the Wilcoxon matched-pairs signed-rank test, which showed that the expression of MTUS1 was downregulated in CRC tissues (Fig. 1D). In addition, the GSE23878 dataset was downloaded to verify the above results, and the results illustrated that MTUS1 was prominently downregulated in CRC tissues compared with normal tissues (Fig. 1E). To further validate the mRNA level of MTUS1, we performed qPCR on our recruited cohort. Not surprisingly, the results again validated the downregulation of MTUS1 in tumour samples (Fig. 1F).

### Relationship between MTUS1 expression and CRC clinicopathological parameters

**Fig. 2:**
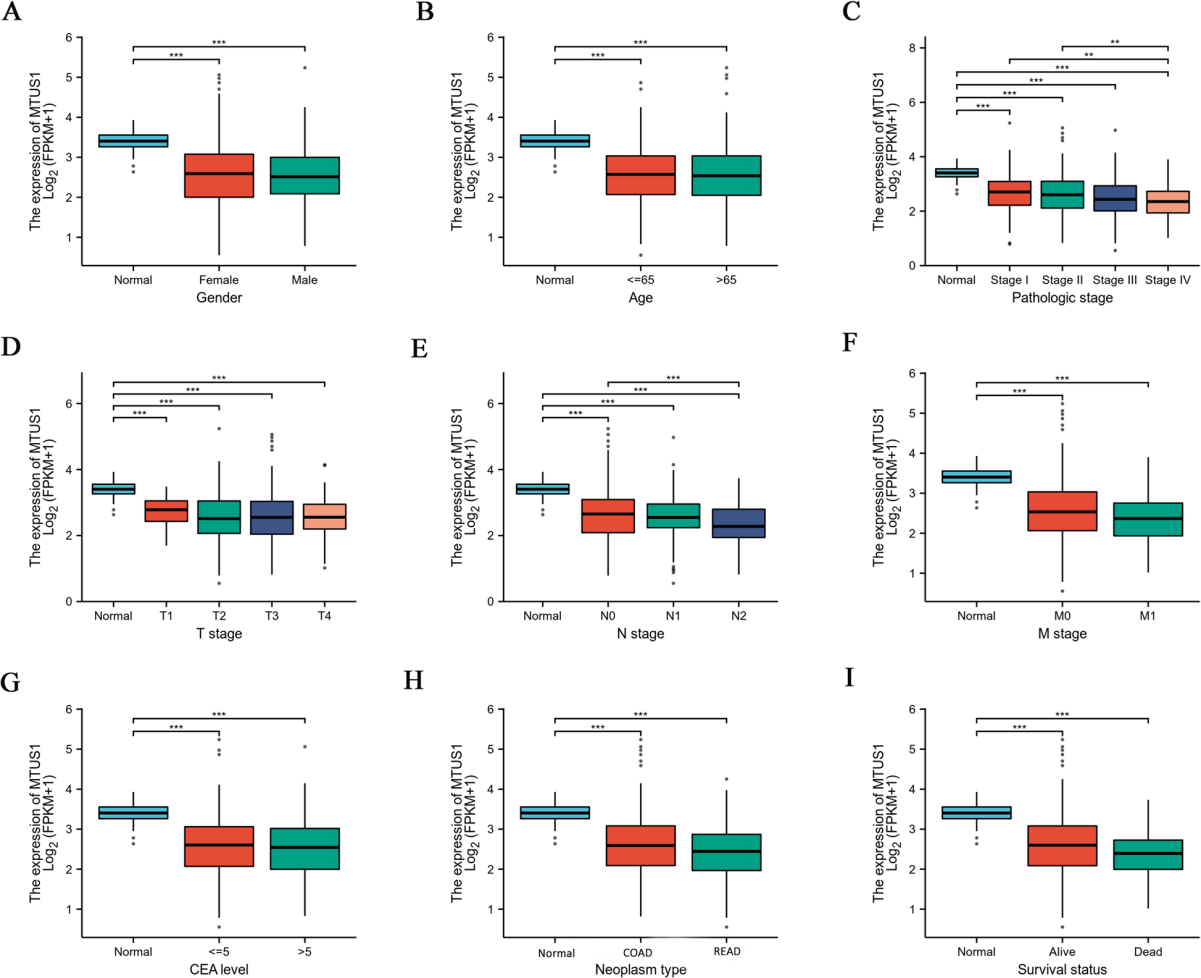
Differential expression analyses for **A** sex, **B** age, **C** pathological stage, **D** T stage, **E** N stage, **F** M stage, **G** CEA levels, **H** neoplasm type, and **I** survival status

Since the expression of MTUS1 was markedly downregulated in CRC tissues, we further analysed the relationship between MTUS1 mRNA expression profiles and clinicopathological parameters using data from 643 CRC patients retrieved from the TCGA database in September 2021 (Table 1). The expression of MTUS1 was significantly decreased in CRC tissues compared to normal tissues, according to the results based on clinicopathological factors, including sex, age, pathologic stage, T stage, N stage, M stage, CEA level, neoplasm type, and survival status (Fig. 2A-I). The expression of MTUS1 decreased in tumour grade IV compared to grades I and II (Fig. 2C). Regarding nodal metastasis status, MTUS1 expression was significantly downregulated in N2 compared to N0 (Fig. 2E). Furthermore, we used logistic regression to investigate the connection between MTUS1 expression and the clinicopathologic features of CRC patients. The patients were divided into high and low MTUS1 expression groups using the median expression level (50%) as the cut-off. CRC patients with lower MTUS1 expression were strongly associated with pathological stage (stage III/IV vs. stage I/II, OR = 0.626, *p* = 0.004), N stage (N1/N2 vs. N0, OR = 1.67, *p* = 0.008), and neoplasm type (READ vs. COAD, OR = 0.699, *p* = 0.048) (Table 2). These results revealed that lower expression of MTUS1 promoted tumour progression and lymph node and distant metastases in CRC patients. Additionally, MTUS1 might have diagnostic and prognostic implications.

**Table 1:** The relationship between MTUS1 mRNA expression profiles and clinicopathological parameters using data from 643 CRC patients retrieved from the TCGA database

Characteristic	levels	Overall
n		644
Age	<=65	276 (42.9%)
	>65	368 (57.1%)
Gender	Female	301 (46.7%)
	Male	343 (53.3%)
T stage	T1	20 (3.1%)
	T2	111 (17.3%)
	T3	436 (68%)
	T4	74 (11.5%)
N stage	N0	368 (57.5%)
	N1	153 (23.9%)
	N2	119 (18.6%)
M stage	M0	475 (84.2%)
	M1	89 (15.8%)
Pathologic stage	Stage I	111 (17.8%)
	Stage II	238 (38.2%)
	Stage III	184 (29.5%)
	Stage IV	90 (14.4%)
CEA level	<=5	261 (62.9%)
	>5	154 (37.1%)
Neoplasm type	Colon adenocarcinoma	478 (74.2%)
	Rectum adenocarcinoma	166 (25.8%)
OS event	Alive	515 (80%)
	Dead	129 (20%)

**Table 2:** Association with pathological stage of CRC patients with lower MTUS1 expression

Characteristics	Total(N)	Odds Ratio(OR) forMTUS1 expression	*P* value
Gender (Male vs. Female)	644	0.809 (0.593-1.102)	0.180
Age (>65 vs. <=65)	644	0.904 (0.661-1.235)	0.524
T stage (T3&T4 vs. T1&T2)	641	1.047 (0.713-1.539)	0.815
**N stage (N1&N2 vs. N0)**	640	0.653 (0.476-0.895)	**0.008**
M stage (M1 vs. M0)	564	0.668 (0.418-1.055)	0.086
**Pathologic stage (Stage III & Stage IV vs. Stage I & Stage II)**	623	0.626 (0.454-0.860)	**0.004**
CEA level (>5 vs. <=5)	415	0.886 (0.594-1.320)	0.552
**Neoplasm type (READ vs. COAD)**	644	0.699 (0.489-0.996)	**0.048**

### Associations between MTUS1 expression and the survival prognosis of CRC patients

**Fig. 3:**
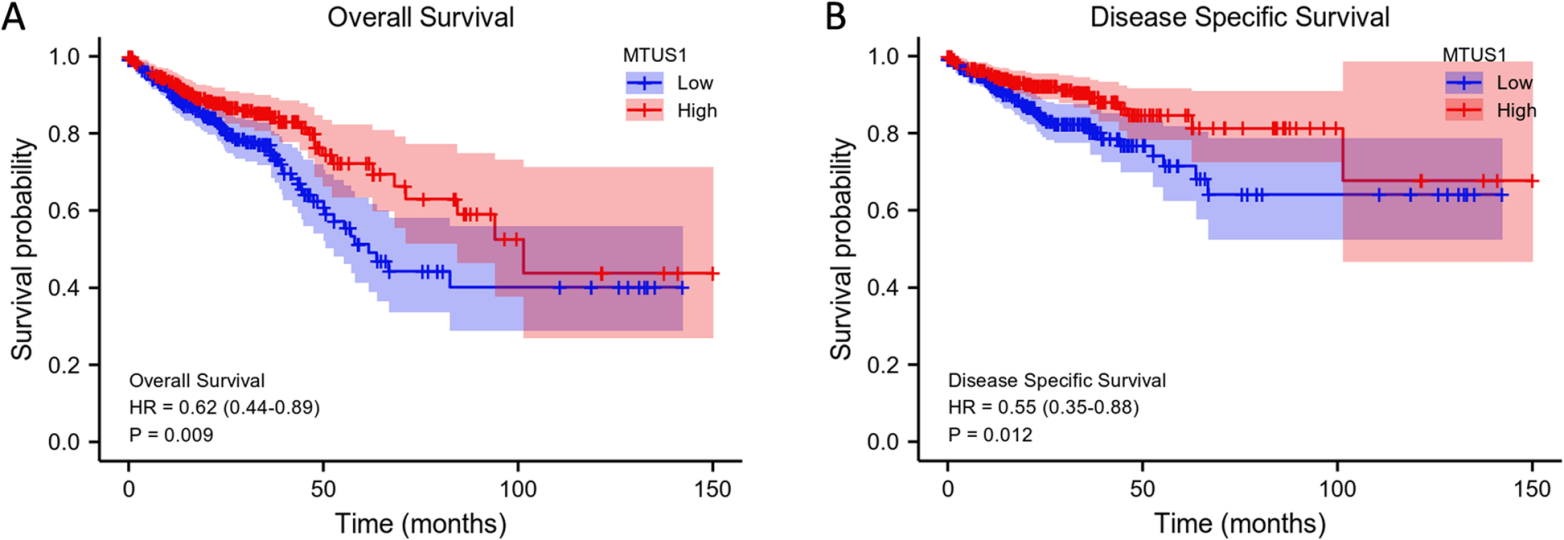
Prognostic value of MTUS1. Kaplan–Meier survival curves comparing **A** OS and **B** DSS between the high and low MTUS1 expression groups

**Fig. 4:**
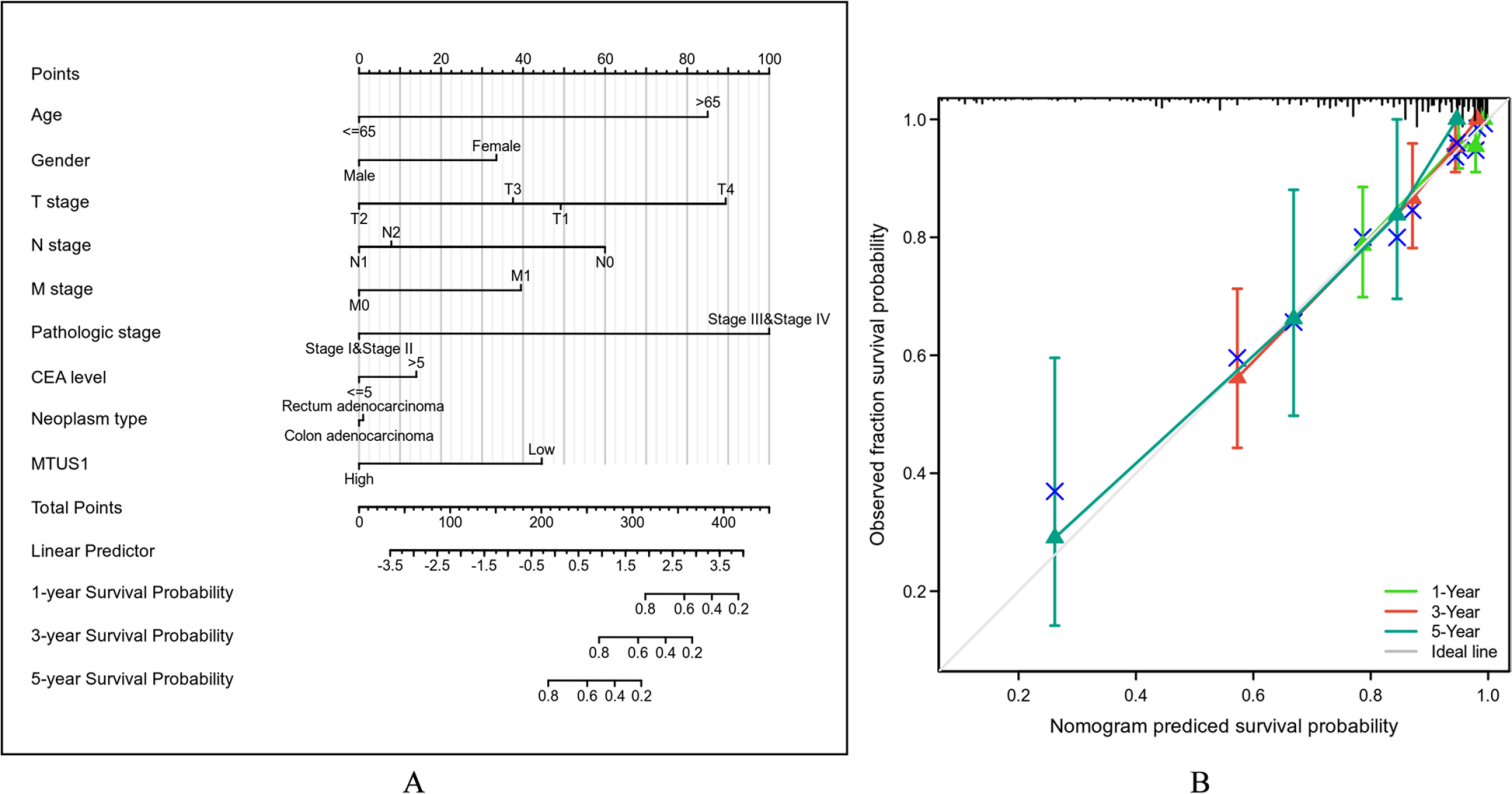
**A** A nomogram was established to predict the survival probabilities of CRC patients at 1, 3, and 5 years based on multifactor regression analyses. **B** The calibration plots showed the good performance of this prognostic model

Furthermore, to identify the prognostic potential of MTUS1 for CRC patients, Kaplan–Meier survival analyses were performed using data from TCGA. Lower expression of MTUS1 was positively correlated with poor OS (HR = 0.62, *p* = 0.009; Fig. 3A) and disease-specific survival (HR = 0.55, *p* = 0.012; Fig. 3B). The univariate Cox regression analysis also demonstrated that low MTUS1 expression was correlated with poor OS [*p* = 0.009, HR = 0.625, 95% CI (0.439–0.890)] (Table 3). Considering other clinicopathologic factors, age, pathologic stage, T stage, N stage, M stage, and CEA level were strongly associated with OS (Table 3). In the multivariate Cox regression analysis, downregulation of MTUS1 and higher pathological stage were independent prognostic factors for poor outcomes (Table 3). Based on this Cox model, we also constructed a nomogram to predict the survival probabilities at 1, 3, and 5 years (Fig. 4A). The calibration plots used to verify the reliability of this prognostic model are shown in Fig. 4B.

**Table 3:**
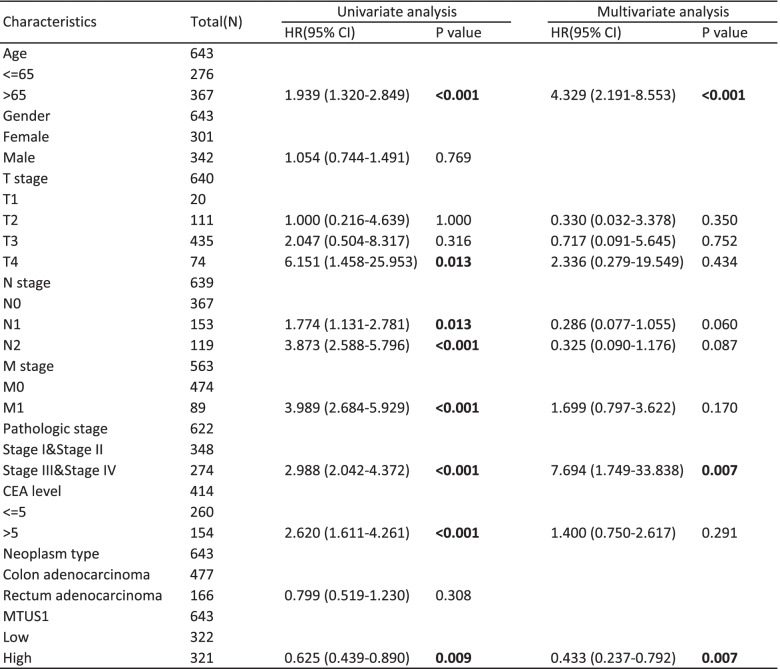
The univariate and multivariate Cox regression analysis

### Diagnostic value of MTUS1 for CRC patients

**Fig. 5:**
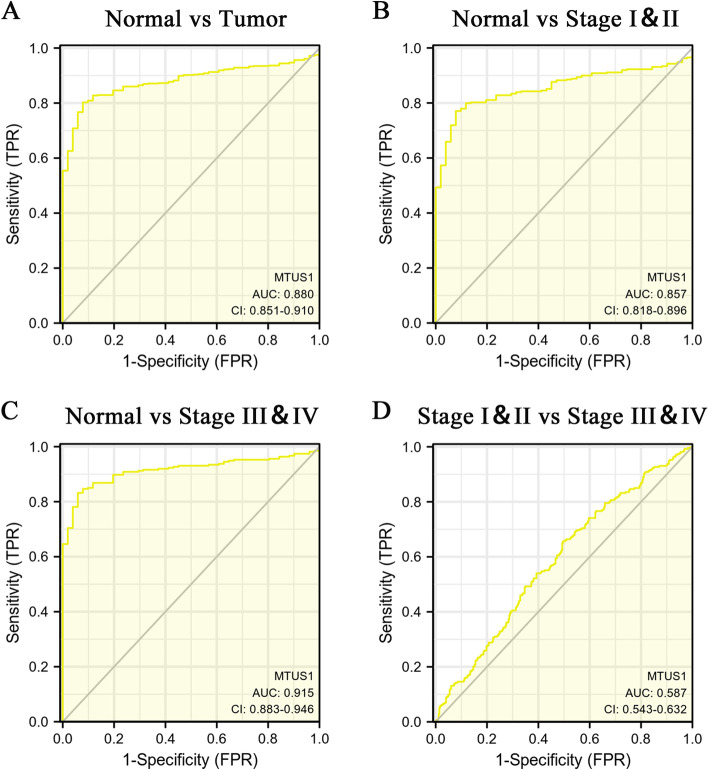
Receiver operating characteristic (ROC) curves of MTUS1 expression in normal vs. cancerous tissues overall (**A**), normal vs. stage I and II cancerous tissues (**B**), normal vs. stage III and IV cancerous tissues (**C**), and stage I and II vs. stage III and IV cancerous tissues (**D**)

The above results showed that the expression of MTUS1 is significantly different in tumour tissues compared to nontumor tissues and that MTUS1 is an independent prognostic factor in CRC patients. Since lower expression of MTUS1 was correlated with poor outcomes, we constructed ROC curves and computed their AUCs to analyse the diagnostic value of MTUS1 for CRC. In this case, larger AUCs indicate higher diagnostic values. The expression of MTUS1 had a modest diagnostic value for all patients (AUC = 0.880; Fig. 5A) and stage I and II patients (AUC = 0.857; Fig. 5B). Meanwhile, MTUS1 demonstrated a high diagnostic value for stage III and IV patients (AUC = 0.915; Fig. 5C). MTUS1 presented a low diagnostic value for all pathological stages (AUC = 0.587; Fig. 5D). Altogether, these results demonstrated that MTUS1 can be used to diagnose CRC in the general population and is a promising diagnostic marker.

### Identification and enrichment analyses of key MTUS1-interacting genes and proteins

**Fig. 6:**
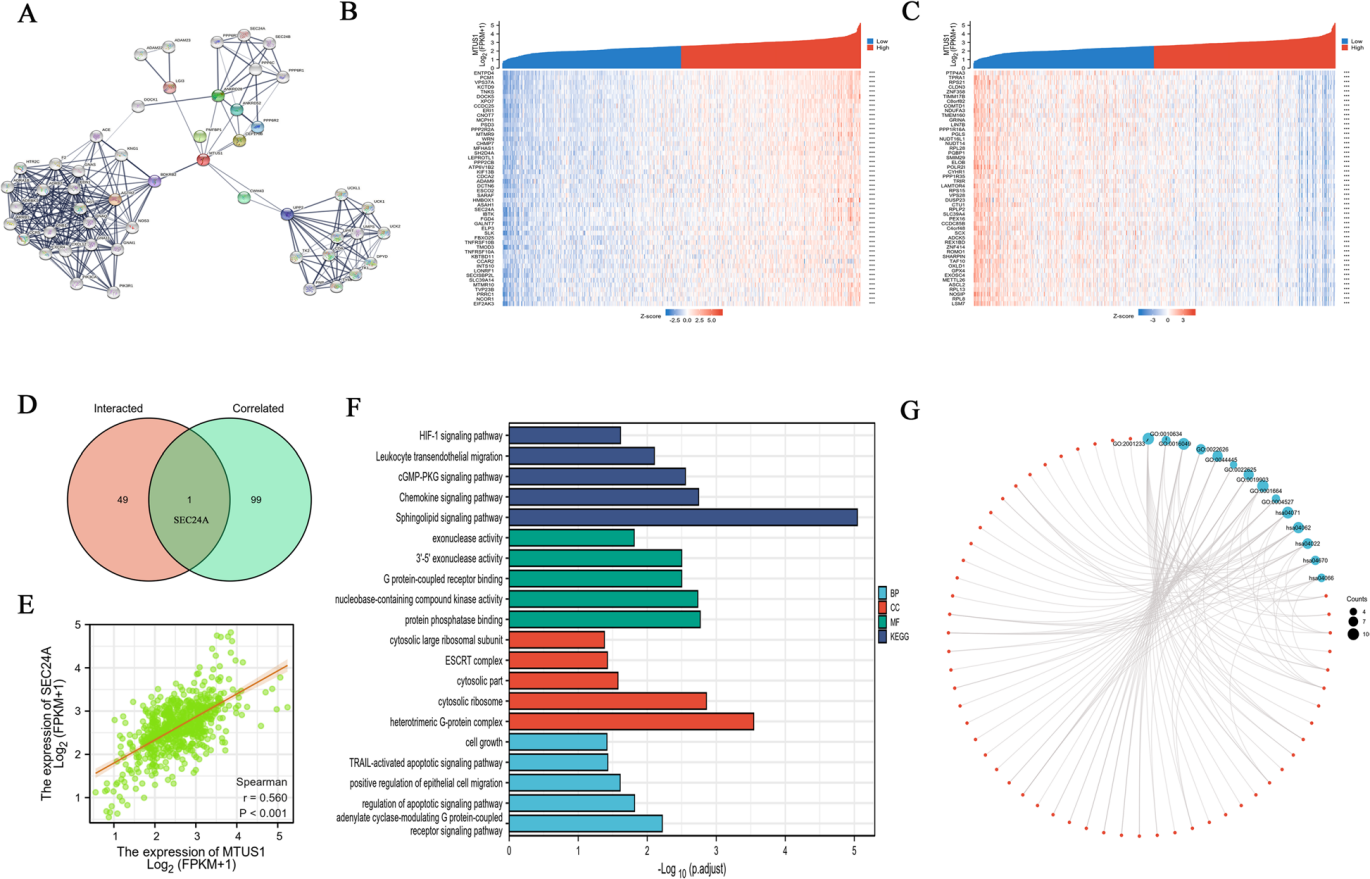
Enrichment analyses of MTUS1-related genes. **A** The top 50 MTUS1-binding proteins were determined by the STRING database. We also obtained the top 100 positively (**B**) and negatively (**C**) coexpressed genes with MTUS1 in TCGA. **D**, **E** Intersection analyses of MTUS1-binding and correlated genes. **F** MTUS1-binding and interacting genes were analysed using GO and KEGG pathway enrichment analyses. **G** Molecular interaction networks according to functional clustering

Furthermore, to investigate the mechanisms of MTUS1 in CRC, we identified key MTUS1-related genes and performed pathway enrichment analyses. The PPI network containing 51 nodes and 280 edges for MTUS1 was constructed using the STRING database (Fig. 6A). The top 10 genes most significantly associated with MTUS1 were AGTR2, CEP170B, ANKRD28, PMFBP1, CWH43, ANKRD52, PPP6R2, UPP2, BDKRB2, and LGI3. Subsequently, we selected the top 100 genes that were correlated with MTUS1 expression in the CRC cohort. The top 50 positively and negatively correlated genes are shown in a heatmap (Fig. 6B, C). Based on these two datasets, we performed a cross-analysis and obtained a common gene, SEC24A (Fig. 6D). Then, we assessed the relationship between MTUS1 and SEC24A using Spearman’s correlation coefficients (Fig. 6E). The GO and KEGG pathway enrichment analyses of these two datasets revealed that these 150 genes are involved in different cancer-associated pathways and biological processes (Fig. 6F, G). KEGG pathways included the HIF-1 signalling pathway, leukocyte transendothelial migration, the cGMP-PKG signalling pathway, the chemokine signalling pathway, and the sphingolipid signalling pathway. GO_BP (biological process) was mainly associated with cell growth, the TRAIL-activated apoptotic signalling pathway, positive regulation of epithelial cell migration, regulation of the apoptotic signalling pathway, and the adenylate cyclase-modulating G protein-coupled receptor signalling pathway. GO_MF (molecular function) was mainly related to exonuclease activity, 3′-5′ exonuclease activity, G protein-coupled receptor binding, nucleobase-containing compound kinase activity, and protein phosphatase binding. Finally, the GO_CC (cellular component) detected terms were cytosolic large ribosomal subunit, ESCRT complex, cytosolic part, cytosolic ribosome, and heterotrimeric G-protein complex. Overall, these results revealed that MTUS1 and MTUS1-related genes were involved in biological processes associated with the onset and progression of tumours, such as immune cell infiltration, tumour cell proliferation, cell migration, and cell apoptosis.

### MTUS1 expression is correlated with immune cell infiltration levels in CRC

**Fig. 7:**
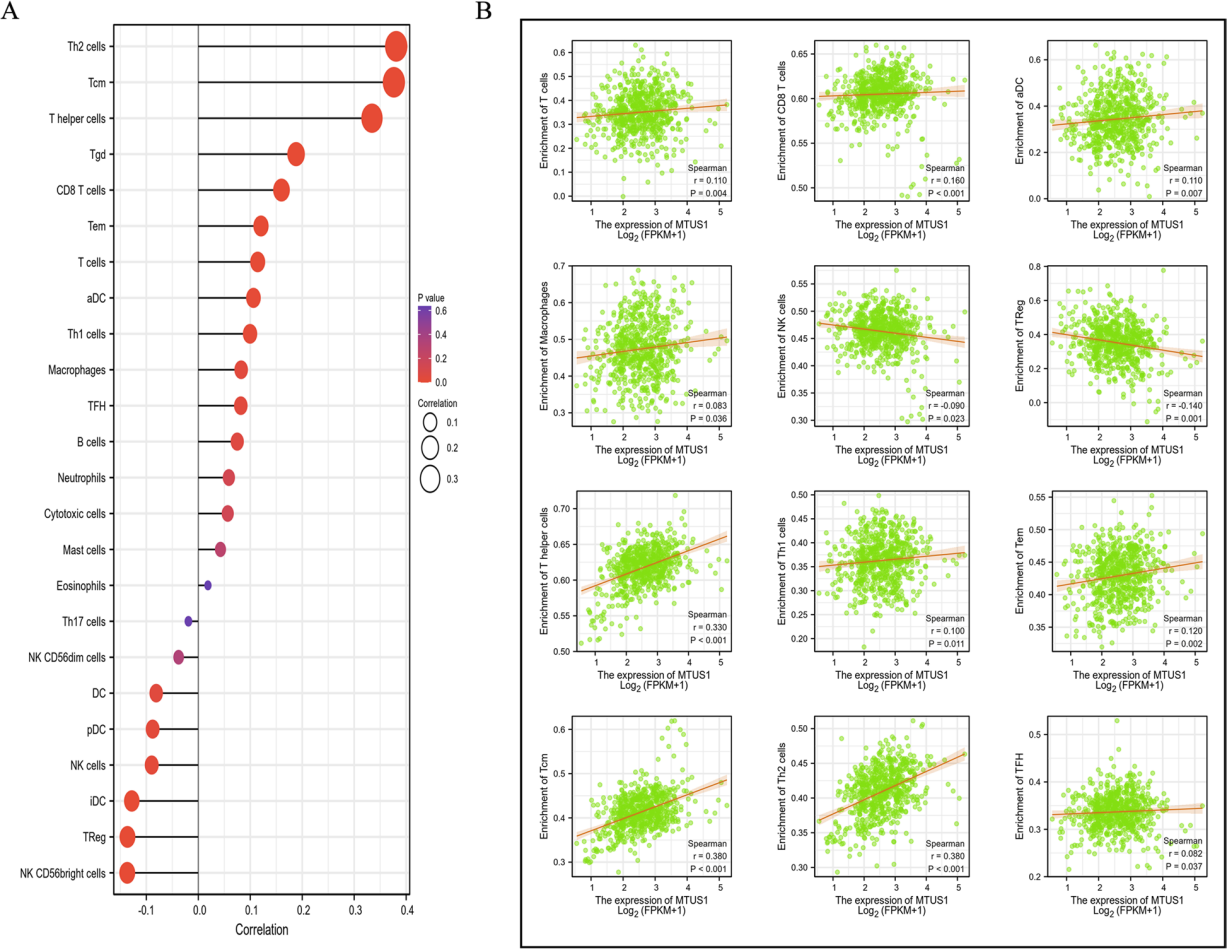
Associations between MTUS1 expression and immune cell infiltration levels by ssGSEA. The results are presented as a lollipop diagram (**A**) and scatter plots (**B**)

**Fig. 8:**
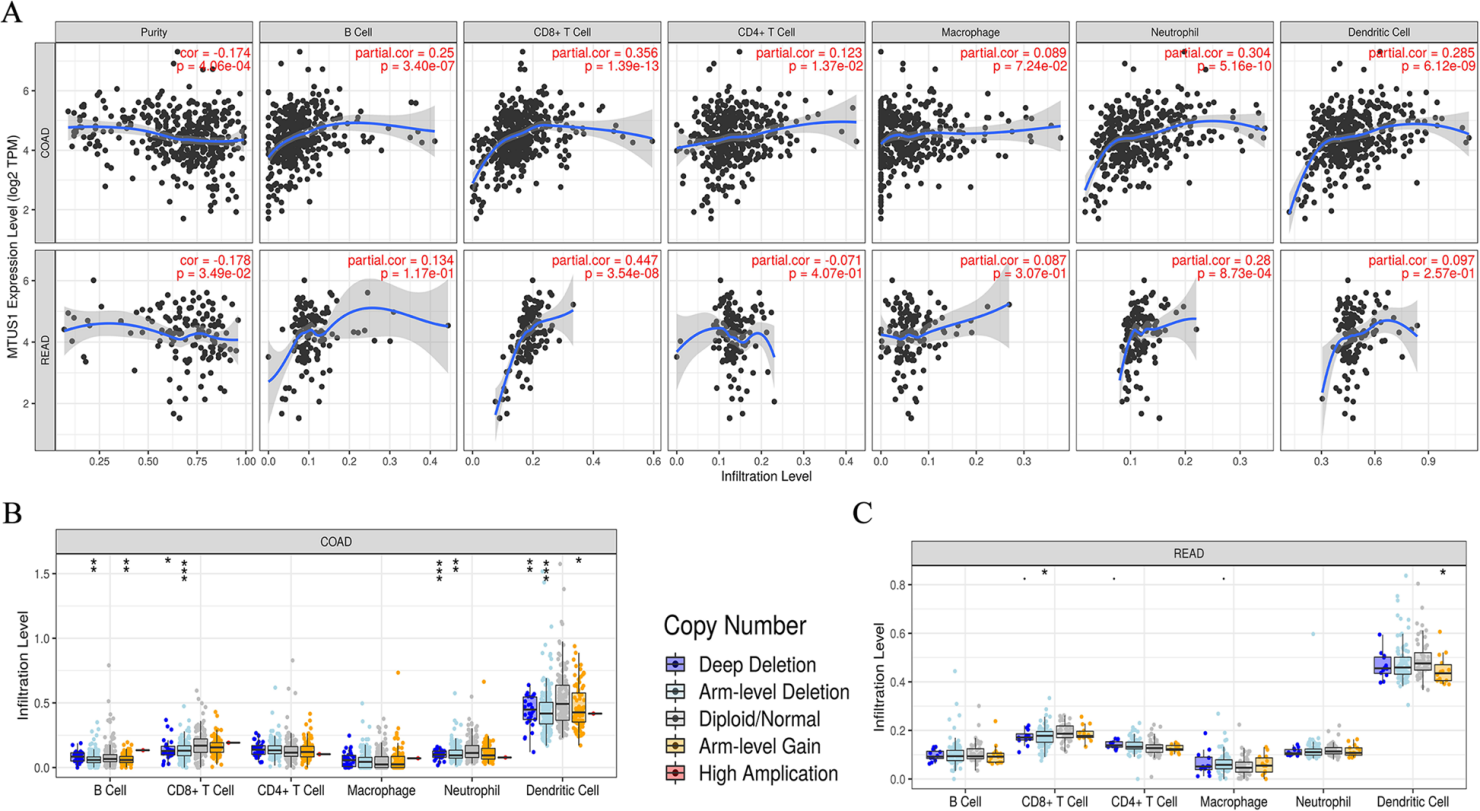
Correlations between MTUS1 expression and immune B cells, CD4+ T cells, CD8+ T cells, neutrophils, macrophages, and dendritic cells in COAD and READ by TIMER (**A**). We also identified the impact of MTUS1 copy number variations on the infiltration levels of six immune cell types in COAD (**B**) and READ (**C**)

Previous studies have demonstrated that the density of TILs within a tumour is an independent predictor of favourable disease-free survival and overall survival [18–20]. Therefore, we assessed the relationship between MTUS1 expression and the infiltration degree of various immune cells using ssGSEA (Fig. 7A). The expression of MTUS1 was significantly and positively correlated with T cells, CD8 T cells, activated dendritic cells, macrophages, T helper cells, Th1 cells, Th2 cells, central memory T (Tcm) cells, effector memory T (Tem) cells, and T follicular helper (TFH) cells. In addition, MTUS1 can negatively regulate the infiltration of natural killer (NK) cells and regulatory T (Treg) cells. Next, to better understand the roles of MTUS1 in CRC, we investigated the relationship between MTUS1 expression and immune infiltration. We also analysed whether the copy number variation of MTUS1 is related to the infiltration levels of immune cells using TIMER. We found that MTUS1 expression was significantly correlated with tumour purity and the infiltration levels of CD8+ T cells and neutrophils in both COAD and READ. MTUS1 was also related to the immune infiltration of B cells, CD4 T cells, macrophages, and dendritic cells in COAD (Fig. 8A). Moreover, the copy number variation of MTUS1 had different degrees of correlation with the infiltration levels of the six immune cell types (Fig. 8B, C). These results suggested that MTUS1 was involved in the recruitment of immune cells. Thus, to confirm the correlation between MTUS1 expression and immune infiltrating cells in CRC, we analysed the immune markers of T cells, CD8+ T cells, B cells, monocytes, TAMs, M1 and M2 macrophages, neutrophils, NK cells, dendritic cells, Th1 cells, Th2 cells, Th17 cells, TFH cells, Tregs, and T-cell exhaustion using the GEPIA web tool. MTUS1 expression was significantly correlated with the expression of CD8A and CD8B in CD8+ T cells; CD3D, CD3E, and CD2 in T cells; CD79A in B cells; CD86 and CSF1R in monocytes; CD68 in TAMs; NOS2 in M1 macrophages; KIR2DL1, KIR2DL3, KIR2DL4, KIR3DL1, KIR3DL2, and KIR3DL3 in NK cells; TBX21, STAT4, STAT1, and IFNG in Th1 cells; GATA3, STAT6, STAT5A, and IL13 in Th2 cells; FOXP3, CCR8, and TGFB1 in Tregs; and PDCD1, CTLA4, LAG3, and HAVCR2 in T-cell exhaustion (all *p* < 0.05) (Table 4).

**Table 4:**
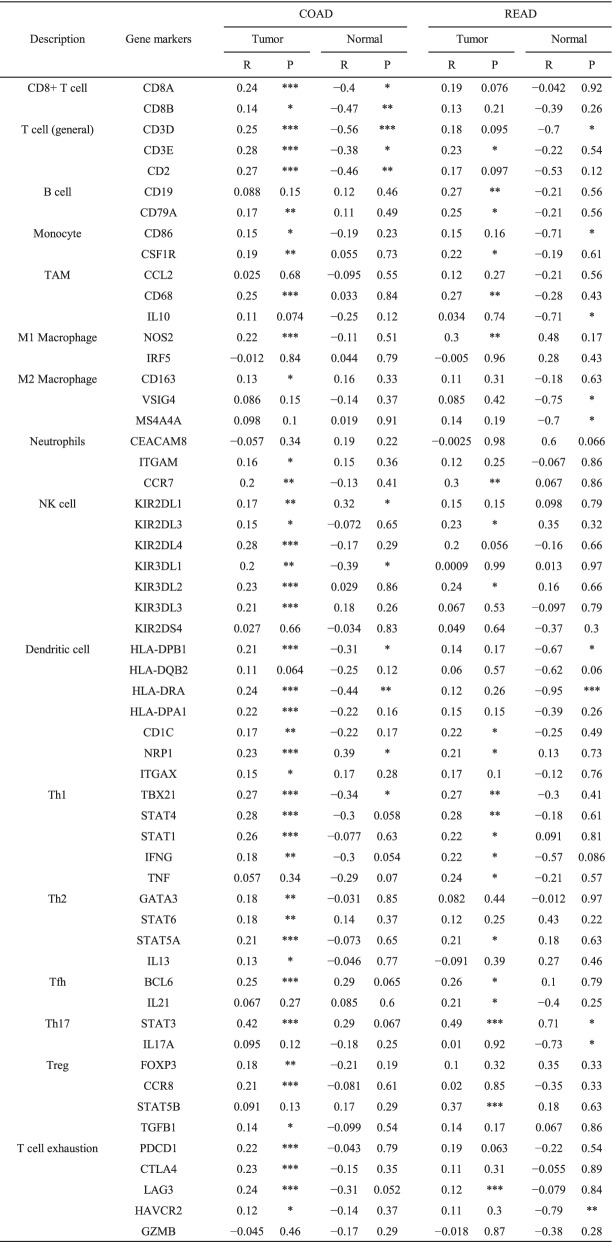
MTUS1 expression

## Discussion

Downregulation of MTUS1 has been previously reported in various cancers, including CRC. However, the diagnostic and prognostic value of MTUS1 in CRC has not yet been investigated. Hence, in the present study, we performed a comprehensive analysis of CRC data from public databases to explore the roles of MTUS1 expression in the survival, prognosis, coexpression network, and immune infiltration of CRC patients. First, the expression of MTUS1 was downregulated in CRC tissues compared with normal control tissues, consistent with the findings of previous studies. Moreover, the expression of MTUS1 was associated with sex, age, pathological grade, TNM stage, CEA level, neoplasm type, and survival status. Then, we explored whether the expression of MTUS1 was correlated with the prognosis of CRC patients. Low expression levels of MTUS1 were significantly correlated with worse OS and DSS, which indicated that these patients had a trend of high survival risk. Logistic regression showed that the expression of MTUS1 in CRC was associated with advanced pathological stages and N stages, suggesting that MTUS1 is important for tumour invasion and lymph node metastasis. The metastatic dissemination of the primary disease is responsible for most cancer-associated mortality, which would explain why low expression of MTUS1 can lead to a poor prognosis in CRC patients. Moreover, the Cox model confirmed that low expression of MTUS1 was an independent indicator of poor prognosis. Next, we constructed a nomogram that presented good performance in predicting the survival probabilities at 1, 3, and 5 years. Additionally, ROC curves demonstrated the potential diagnostic value of MTUS1. Then, to explore the molecular mechanisms and functions of MTUS1 in CRC pathogenesis, we performed KEGG pathway and GO enrichment analyses based on MTUS1-interacting proteins and coexpressed genes. We found that the proteins interacting with MTUS1 were involved in multiple pathways related to cancer development. Furthermore, we provided evidence that the levels of immune infiltrates and diverse immune marker sets were associated with MTUS1 expression levels, which might extend the understanding of the roles of MTUS1 in tumour immunology.

Previous studies have shown that the expression of MTUS1 is correlated with different cell phenotypes, such as proliferation, differentiation, apoptosis, and ubiquitination, and is a prognostic indicator for multiple cancers. MTUS1 can also regulate cell division by disturbing the microtubule cytoskeleton and can also be used to predict the treatment response to paclitaxel-based chemotherapy in breast cancer [21–23]. In lung adenocarcinoma tissues, low MTUS1 expression is related to various clinical-pathological parameters, such as tumour size, Ki-67 proliferation index, lymphovascular invasion, and lymph node metastasis, which lead to the correspondingly poor prognosis of patients [14]. Moreover, MTUS1 expression levels can be synergistically inhibited by miR-19a and miR-19b, thereby contributing to lung cancer cell proliferation and migration [24]. Furthermore, the functional effect and prognostic value of low MTUS1 expression in other cancers were successively confirmed [10, 15, 17, 25, 26]. Although the downregulation of MTUS1 and its tumour-suppressor function have also been previously reported in CRC, the specific molecular mechanisms underlying MTUS1 expression remain to be elucidated [27, 28]. In our current study, KEGG pathway enrichment analyses were performed with MTUS1 coexpressed genes in CRC and showed an association with the HIF-1 pathway. The HIF-1 pathway mediates cell proliferation and apoptosis [29, 30], which might explain why high MTUS1 expression is correlated with a better prognosis in cancer patients.

Furthermore, the relationship between the expression of MTUS1 and tumour-infiltrating immune cells has not been previously reported. The status and density of tumour-infiltrating lymphocytes can predict cancer prognosis and are related to tumorigenesis and tumour progression, thereby playing a critical role in antitumor immune therapies [31–33]. Thus, the unique feature of our current study was the comprehensive evaluation of the relationship between MTUS1 expression and typical markers of different immune cell types, its potential impact on the recruitment of immune cells to the tumour microenvironment, and the potential immune-related mechanisms mediated by MTUS1. Immune infiltration analysis using ssGSEA showed a significant positive correlation between MTUS1 expression and the infiltration levels of T cells, Th1 cells, Th2 cells, central memory T (Tcm) cells, T helper cells, and CD8 T cells. Meanwhile, a negative correlation between MTUS1 expression and the infiltration levels of Tregs and NK cells was detected. Similarly, we found that MTUS1 expression levels were correlated with the infiltration degrees of B cells, CD4+ T cells, CD8+ T cells, neutrophils, macrophages, and dendritic cells in the TIMER database. These correlations suggested that MTUS1 can recruit immune T, Th1, Th2, Tcm, T helper, CD4+ T, and CD8+ T cells into the tumour microenvironment and prevent the recruitment of Treg cells that promote immune tolerance and angiogenesis. CD8+ T cells can search for tumour antigens and directly kill tumour cells, which is crucial in the adaptive immune response against cancers [34–36]. Th1 and dendritic cells can also mediate cellular immune responses against tumours [37–39]. Moreover, NK cells and neutrophils participate in the antitumor immune response [40, 41]. On the other hand, although Treg cells are also required to control immune responses and maintain tumour microenvironment homeostasis, they can suppress antitumor immune responses contributing to tumour immune evasion [42, 43]. The results from the expression correlation analysis using GEPIA showed that MTUS1 expression levels in tumour tissues, especially CRC tissues, were closely related to most marker sets of immune cells, consistent with the findings of these previous studies.

The above findings suggested that MTUS1 can play a key role in the regulation of immune cell infiltration and is a CRC immune-modulating factor. Previous studies have reported that the interaction between the lncRNA LIFR-AS1 and MTUS1 can block the MEK/ERK pathway, thereby inhibiting tumour cell proliferation, migration, and invasion [16, 44]. Furthermore, the repression of the MEK/ERK pathway can change the immune cell composition of the tumour immune microenvironment [45]. Hence, MEK/ERK pathway inhibition by MTUS1 can be a potential mechanism for the upregulation of immune infiltrates in CRC. Nevertheless, further in-depth exploration is needed to understand the precise functions and mechanisms of MTUS1 in the tumour immune microenvironment. Our current study provides new insights into cancer immunotherapy. However, it also has some limitations. First, we only performed bioinformatic analyses using patient data from the TCGA database. Hence, it is difficult to judge the true impact of MTUS1 on development-related signalling pathways and biological behaviour. Therefore, more experiments are needed to verify the expression and related mechanisms of MTUS1 and elucidate its true associations with tumour-infiltrating immune cells.

Overall, the expression of MTUS1 was significantly decreased and was correlated with the clinicopathological stages and prognosis of CRC patients. Moreover, MTUS1 and its associated pathways are involved in CRC development and progression. Additionally, the expression of MTUS1 might affect immune cell infiltration levels. Therefore, MTUS1 is a meaningful diagnostic and sensitive prognostic marker for CRC and is involved in the infiltration of immune cells in the tumour microenvironment. Finally, we provided a promising direction for CRC clinical diagnosis and treatment.

## Data Availability

The datasets supporting the conclusion of this article are included within the article.
